# Radiographic Features and Clinical Factor for Preoperative Prediction in the Bulging Duodenal Papilla With Malignancy

**DOI:** 10.3389/fonc.2021.627482

**Published:** 2021-04-01

**Authors:** Xiao-Jie Wang, Jun-Li Ke, Jian-Xia Xu, Jia-Ping Zhou, Yuan-Fei Lu, Qiao-Mei Zhou, Dan Shi, Ri-Sheng Yu

**Affiliations:** ^1^ Department of Radiology, Second Affiliated Hospital, Zhejiang University School of Medicine, Hangzhou, China; ^2^ Department of Radiology, The Second Affiliated Hospital of Zhejiang Chinese Medical University, Hangzhou, China

**Keywords:** duodenal papilla, computed tomography, preoperative prediction, direct bilirubin, scoring system

## Abstract

**Background:**

To investigate characteristic clinical and imaging features and establish a scoring system for preoperative prediction of malignancy in the bulging duodenal papilla.

**Methods:**

A total of 147 patients with bulging duodenal papilla (Benign enlargement n = 67; malignant enlargement n = 80) from our hospital between 2010 and 2020 were retrospectively analyzed. We investigated meaningful clinical and CT imaging features and established the score model through logistic regression and weighted. The calibration test, the ROC, AUC, and cut-off points were performed in score model. The model was also divided into three score ranges for convenient clinical evaluation.

**Results:**

Three clinical and CT imaging features were finally included in the score model including direct bilirubin (DBil) increase >7 umol/L (3 points), pancreatic duct (PD) dilation >5 mm (2 points), and irregular shape (2 points). The AUCs of the primary predictive model and score model were 0.896 (95% CI, 0.835–0.940) and 0.896 (95% CI, 0.835–0.940), respectively. This scoring system presented with a sensitivity of 78.8% and a specificity of 88.1% when using 2.5 points as cutoff value. Three score ranges were also proposed for convenient clinical use as follows: 0–2 points; 3–4 points; 5–7 points. The number of patients with malignant duodenal papillary enlargement increased with the increasing scores.

**Conclusions:**

We proposed a convenient scoring system to preoperative predict malignancy in the bulging duodenal papilla.

## Introduction

The major duodenal papilla is a functional region where the pancreatic duct (PD) and the bile duct enter the duodenum, and the maximal diameter of the size of normal duodenal papilla were 5–10 mm as reported by previous study (1–3). Various pathologic conditions, such as papillitis, diverticulum, benign and malignant tumor (4, 5), can cause bulging papilla that is frequently seen at computed tomography (CT). And it is more difficult to identify the course when there was only enlarging duodenal papilla without obvious lesions in neighboring organization.

Endoscopic retrograde cholangiopancreatography (ERCP) is now used as golden standard to identify the pathologic conditions of the bulging papilla (6, 7). But because of the invasive operation and may be major post-procedural complications, like pancreatitis, hemorrhage, perforation, and even death (6–8), it would be helpful for patients to find a non-invasive and reliable method to predict malignancy of the enlarging duodenal papilla.

CT as one of the most widely used and non-invasive abdominal imaging methods has presented the latent energy to differentiate between benign and malignant bulging papilla as demonstrated by previous imaging study. Lobular masses, dilatation of the common bile duct, PD, intra- and extrahepatic bile duct, and so on were reported as meaningful indication (9, 10). However, it may be not reliable to depend such a few CT features with ignorance of other clinical characteristics to diagnose malignancy in bulging duodenal papilla.

Therefore, this study aims to investigate independent clinical and CT imaging risk characteristics, and then establish a convenient scoring system for preoperative prediction of malignancy in bulging duodenal papilla.

## Methods

### Patients

**Figure 1:**
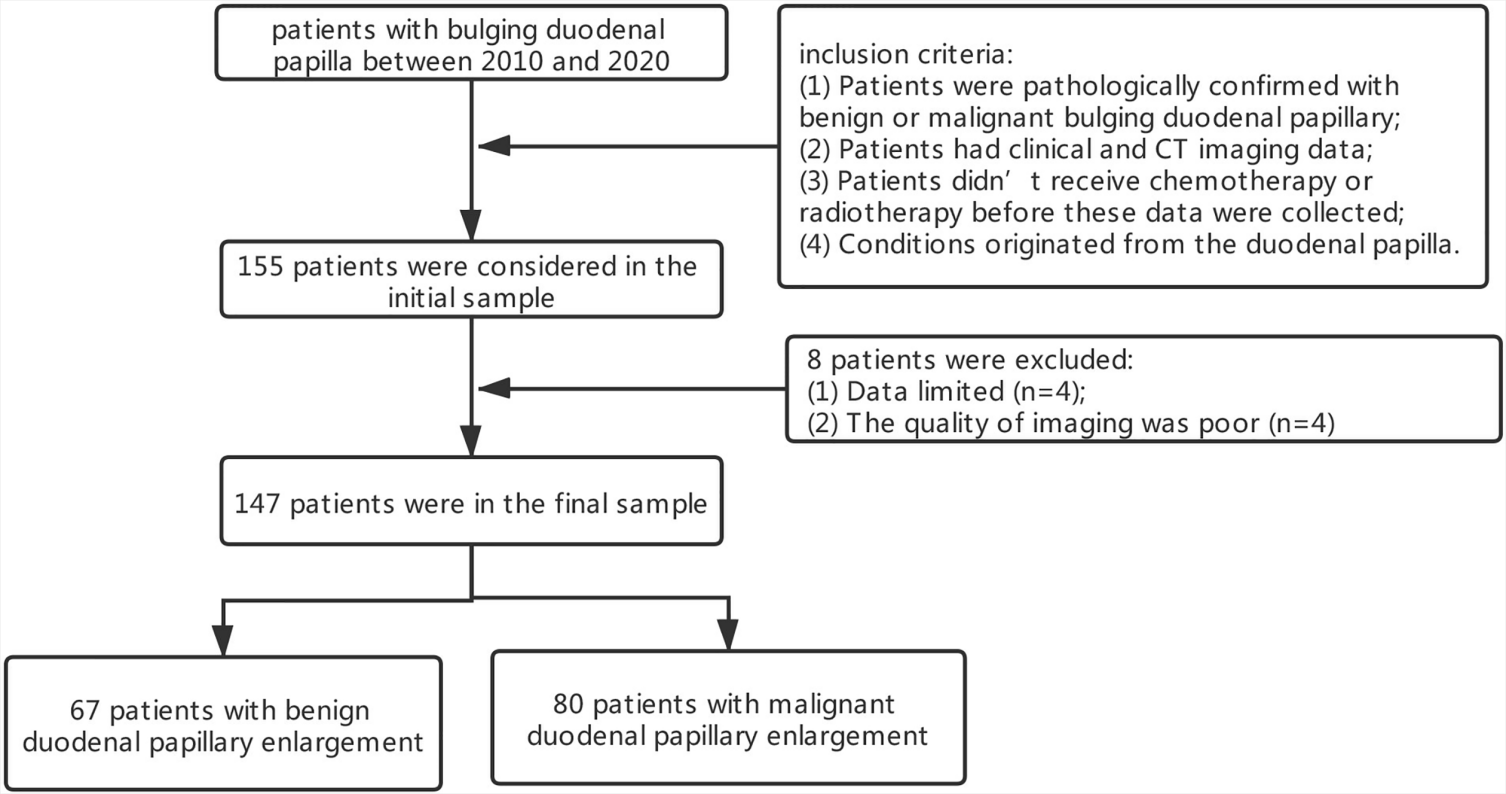
Patient flow diagram.

Our institutional review board approved this retrospective study and waived consent requirement from patients. A total of 147 patients were finally included in this study population through searching the medical records from 2010 to 2020 in the Second Affiliated Hospital of Zhejiang University School of Medicine according to the following inclusion criteria: (1) patients were pathologically confirmed with benign or malignant bulging duodenal papillary; (2) patients had clinical and CT imaging data; (3) Patients didn’t receive chemotherapy or radiotherapy before these data were collected; (4) conditions originated from the duodenal papilla. Eight patients were excluded because of the following reasons: (1) Data limited (n = 4); (2) The quality of imaging was poor (n = 4). The final study cohort was consisted of 67 patients with benign duodenal papillary enlargement (including inflammation, diverticulum, and duodenal papillary adenoma) and 80 with malignant duodenal papillary enlargement (**Figure 1**).

### Data Acquisition

Abdominal contrast-enhanced CT examinations of patients were performed in one multidetector-row CT (SOMATOM Definition Flash; Siemens Healthcare). The scanning parameters were same as follows: detector configuration 128 × 0.6 mm, tube voltage 120 kVp, tube current 200 mAs, slice thickness 5 mm, slice interval 5 mm, pitch of 0.6 mm. A total of 120 ml of contrast agent was administered with a pump injector at 3–4 ml/s into an antecubital vein. The arterial and portal venous phases were obtained at 40–50 s and 80–90 s after the injection of the contrast medium, respectively. The clinical data were collected by screening the institutional medical reports.

### Collection of Clinical Data

All patients were performed with required examination. The clinical data included age, gender, clinical symptoms (abdominal discomfort or jaundice), total bilirubin (TBil) increase (>17.1 umol/L), direct bilirubin (DBil) increase (>7 umol/L), indirect bilirubin (IBil) increase (>13.7 umol/L), and Carbohydrate antigen199 (CA199) increase (>37 Ku/L).

### Analysis of the Images

All the images were evaluated by two experienced abdominal radiologists independently who were unknown of the pathology result. The variables of CT imaging were as follows: The shape of duodenal papilla (regular or irregular), extrahepatic bile duct dilation (EHD) (>10 mm, >20 mm), intrahepatic bile duct dilation (IHD) (>5 mm), PD dilation (>3 mm, >5 mm), asymmetric thicken of the distal of the common bile duct, thicken of the adjacent duodenal wall, target sign, cut off suddenly of the common bile dilation, the CT attenuation of the lesions in three phases, and correlated two ratios. Target sign indicated that dilated common bile duct extended the baseline of the inner wall of the duodenum. Ratio 1 was defined as CT attenuation of arterial phase minus that of plain scanning, ratio 2 was defined as CT attenuation of portal phase minus that of plain scanning.

### Statistical Analysis

Continuous variables are presented as median with standard deviation (M-S), and categorical variables as number with percentage. The same variables between two groups were compared using the Student t test for continuous variables and chi-square or Fisher’s exact test for categorical variables. Variables that presented statistically significant in univariate analysis (P < 0.05) were obtained into ridge regression analysis to minimize multicollinearity (11) and then obtained into a logistic regression model. For the development of an integer-based scoring system, we used the method presented by Ben AH et al. (12), which converted regression coefficients to weight scores through dividing each coefficient with one-half of the smallest beta coefficient and then rounded to the nearest integer. The total score range were calculated through summing the individual score corresponding to the related variables. The receiver operating characteristic curve (ROC) curve and the area under curve (AUC) was performed to evaluate the discriminatory of the models, and Hosmer-Lemeshow was used to access the calibration of the models. Comparison between ROCs of different models was performed through Delong nonparametric method (Delong and others 1988).

All the data were analyzed by SPSS version 25.0 software (IBM Crop, Armonk, NY, USA), except ROCs comparison was performed by MedCalc statistical software, version 19.0 (MedCalc Software Bvba, Ostend). A two-sided p value <0.05 considered statistically significant.

## Results

### Clinical Characteristics in Patients

The comparison of clinical characteristics was summarized in **Table 1**. There was no significant difference between benign and malignant bulging duodenal papilla with regard to age and abdominal discomfort. But gender, jaundice, TBil increase (>17.1 umol/L), DBil increase (>7 umol/L), IBil increase (>13.7 umol/L), and CA199 increase (>37 Ku/L) presented significant difference.

**Table 1:** Clinical characteristics in patients.

	Benign bulging duodenal papilla(n = 67)	Malignant bulging duodenal papilla (n = 80)	P
Gender			0.036
Female	45 (67.2%)	40 (50.0%)	
Male	22 (32.8%)	40 (50.0%)	
Age	61.88 ± 10.73	62.74 ± 9.16	0.602
Abdominal discomfort			0.510
No	32 (47.8%)	43 (53.8%)	
Yes	35 (52.2%)	37 (46.2%)	
Jaundice			<0.001
No	67 (100%)	45 (56.3%)	
Yes	0 (0.0%)	35 (43.7%)	
Total bilirubin (TBil) increase (>17.1 umol/L)			<0.001
No	48 (71.6%)	17 (21.3%)	
Yes	19 (28.4%)	63 (78.7%)	
Direct bilirubin (DBil) increase (>7 umol/L)			<0.001
No	60 (89.6%)	20 (25.0%)	
Yes	7 (10.4%)	60 (75.0%)	
Indirect bilirubin (IBil) increase (>13.7 umol/L)			<0.001
No	51 (76.1%)	23 (28.7%)	
Yes	16 (23.9%)	57 (71.3%)	
CA199 increase (>37 Ku/L)			<0.001
No	60 (89.6%)	45 (56.3%)	
Yes	9 (10.4%)	35 (43.7%)	

### Imaging Features in Patients

The comparison of CT imaging characteristics was summarized in **Table 2**, which presented significant difference in the shape of duodenal papilla, EHD dilation (>10 mm, >20 mm), IHD dilation (>5 mm), PD dilation (>3 mm, >5 mm), and cut off suddenly of the common bile dilation.

**Table 2:** Imaging features in patients.

	Benign bulging duodenal papilla (n = 67)	Malignant bulging duodenal papilla (n = 80)	P
Extrahepatic bile duct dilation (EHD) >10 mm			<0.001
No	14 (20.9%)	1 (1.3%)	
Yes	53 (79.1%)	79 (98.7%)	
EHD >20 mm			<0.001
No	56 (83.6%)	35 (43.8%)	
Yes	11 (16.4%)	45 (56.2%)	
Intrahepatic bile duct dilation (IHD) >5 mm			<0.001
No	41 (61.2%)	10 (12.5%)	
Yes	26 (38.8%)	70 (87.5%)	
The shape of duodenal papilla			0.026
regular	63 (94.0%)	65 (81.2%)	
irregular	4 (6.0%)	15 (18.8%)	
CT attenuation in plain scanning	38.47 ± 4.892	39.95 ± 5.50	0.089
CT attenuation in arterial phase	79.10 ± 19.062	79.4 ± 17.27	0.923
CT attenuation in portal phase	83.54 ± 14.440	84.78 ± 15.98	0.628
Ratio 1	2.07 ± 0.51	2.02 ± 0.50	0.504
Ratio 2	2.20 ± 0.42	2.15 ± 0.44	0.524
Pancreatic duct dilation (PD) >3 mm			0.009
No	43 (64.2%)	34 (42.5%)	
Yes	24 (35.8%)	46 (57.5%)	
PD >5 mm			<0.001
No	61 (91.0%)	47 (58.8%)	
Yes	6 (9.0%)	33 (41.2%)	
Asymmetric thicken of the distal of the common bile duct			0.126
No	67 (100.0%)	76 (95.0%)	
Yes	0 (0.0%)	4 (5.0%)	
Thicken of the adjacent duodenal wall			0.251
No	67 (100.0%)	77 (96.3%)	
Yes	0 (0.0%)	3 (3.7%)	
Target sign			0.053
No	60 (89.6%)	62 (77.5%)	
Yes	7 (10.4%)	18 (22.5%)	
Cut off suddenly of the common bile dilation			<0.001
No	59 (88.1%)	42 (52.5%)	
Yes	8 (11.9%)	38 (47.5%)	

### Development of the Preoperative Predictive Model

In the univariate analysis, totally 13 clinical and CT features showed statistical difference as demonstrated in **Tables 1** and **2** [gender, jaundice, TBil increase (>17.1 umol/L), DBil increase (>7 umol/L), IBil increase (>13.7 umol/L), CA199 increase (>37 Ku/L), the shape of duodenal papilla, EHD dilation (EHB) (>10 mm, >20 mm), IHD dilation (>5 mm), PD dilation (>3 mm, >5 mm), and cut off suddenly of the common bile duct], which then were included in ridge regression analysis to minimize multicollinearity in multivariate analysis. When K value was 0.2, the ridge trace presented with the standardize coefficients of variables was to be stable and the model was significant (P < 0.001), where five variables presented positive correlation with malignant duodenal papillary diagnosis, including DBil increase (>7 umol/L) (P < 0.0001), PD dilation (>5 mm) (P = 0.004), irregular shape (P = 0.048), jaundice (P = 0.02), and IHD dilation (>5 mm) (P = 0.041).

Multivariate logistic regression was performed to get further verification, and three variables showed independent correlation with the diagnosis of malignant duodenal papillary lesions in this primary preoperative predictive model including DBil increase (>7 umol/L) (OR 36.968; 95% CI 12.74–107.277), PD dilation (>5 mm) (OR 8.403; 95% CI 2.509–28.14), and irregular shape (OR 7.435; 95% CI 1.73–31.953), as demonstrated in **Table 3**, which were finally adopted to develop the scoring system. Hosmer-lemeshow goodness-fit test presented good calibration of this primary preoperative predictive model (P = 0.780>0.05), and the AUC of the model was 0.896 (95% CI 0.835–0.940; P < 0.0001).

**Table 3:** Establishment of the scoring system.

	Univariate analysis	Multivariate analysis				
	P	P	HR	95% CI	B	Score
Direct bilirubin (DBil) increase (>7 umol/L) (Yes)	<0.0001	<0.0001	36.968	12.74–107.277	3.610	3
Jaundice (Yes)	0.998					
Intrahepatic bile duct dilation (IHD) >5 mm (Yes)	0.177					
Pancreatic duct dilation (PD) >5 mm (Yes)	0.001	0.001	8.403	2.509–28.14	2.129	2
Irregular shape of papilla (Yes)	0.021	0.007	7.435	1.73–31.953	2.006	2

### Development of the Scoring System

To provide a quantitative method to predict malignant duodenal papillary lesions, a scoring system was proposed based on multivariate analysis. Weighted scores were assigned to three independent variables as follows: DBil increase (>7 umol/L), 3 points; PD dilation (>5 mm), 2 points; irregular shape, 2 points (**Table 3**). After summing the individual score corresponding to the related variables, a scoring system (range from 0 to 7) was finally constructed. Hosmer-lemeshow goodness-fit test presented good calibration of this score model (P = 0.434>0.05), and the AUC of the model was 0.896 (95% CI 0.835–0.940; P < 0.0001), similar to the primary preoperative predictive model. And the comparison of ROCs verified by DeLong test showed no statistical difference between two models (P = 0.9145>0.05), indicating that the score model made full use of the value of the primary predictive model. When use 2.5 points as the cutoff value, the sensitivity of this scoring system was 78.8% and the specificity was 88.1%.

**Figure 2:**
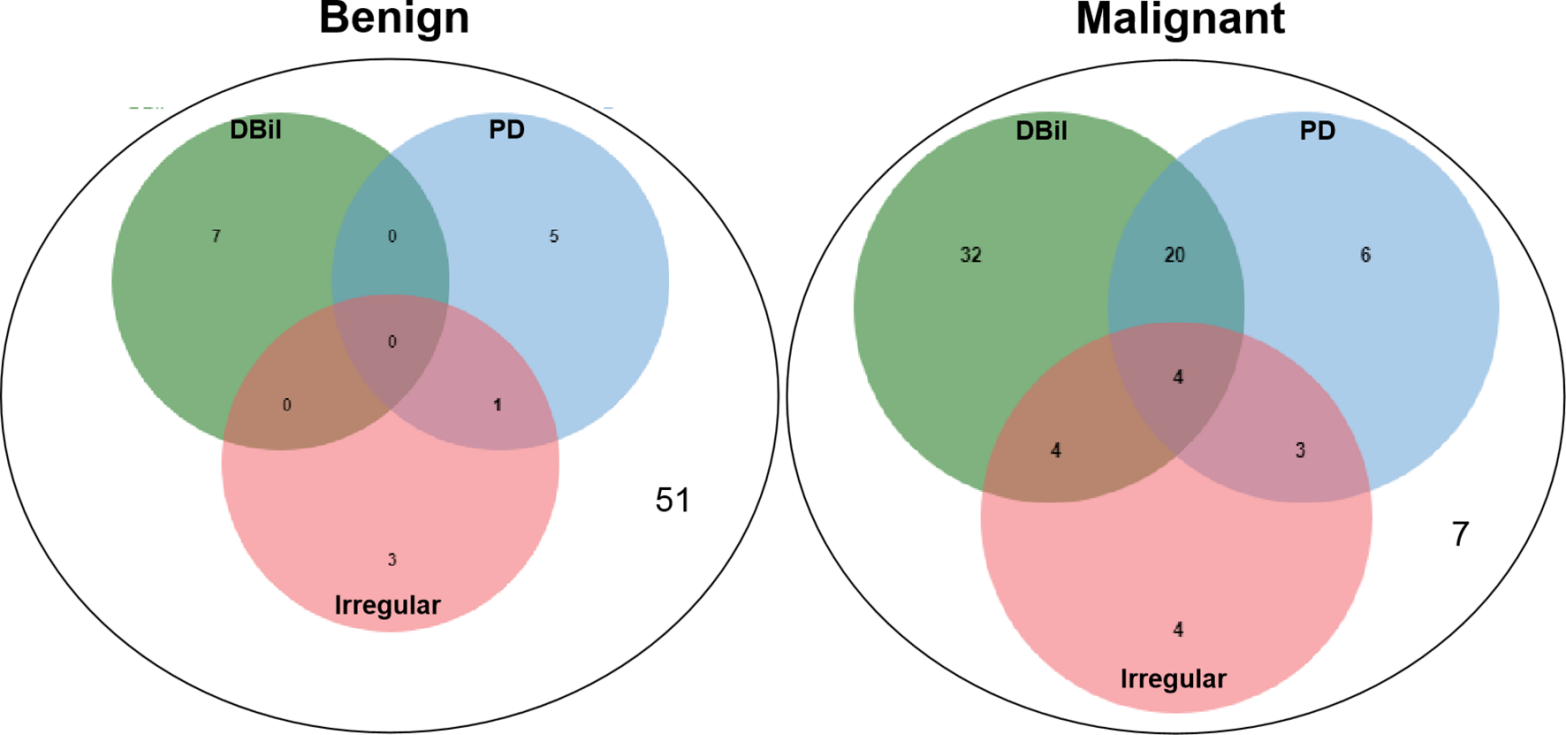
The correlation of the three critical factors and bulging duodenal papilla is presented by a Venn diagram, which performed by Draw Venn Diagram Website (http://jvenn.toulouse.inra.fr/app/example.html).

To apply this scoring system conveniently in practice, we further divided it into three score ranges as follows: 0–2 points; 3–4 points; 5–7 points. The predictive positive rates of the three ranges increased as demonstrated in **Table 4**. The correlation of the three critical factors and bulging duodenal papilla is presented by a Venn diagram (**Figure 2**).

**Table 4:** Patients with malignant bulging duodenal papilla in three score ranges.

Score groups	Number of patients with malignant bulging duodenal papilla	Total Number	Diagnostic probability of malignancy
0–2 points	17	76	about 22.4%
3–4 points	35	43	about 81%
5–7 points	28	28	about 100%

## Discussion

The scoring system established in this study could successfully detect malignant bulging duodenal papilla through observing three clinical and CT imaging features, including DBil increase (>7 umol/L), PD dilation (>5 mm), and irregular shape of the papilla (**Table 5**).

**Table 5:** The scoring system for preoperative prediction in the bulging duodenal papilla with malignancy.

Evaluation factors	Score	Risk-Total Score
Direct bilirubin (DBil)		Low risk: 0–2 points; high risk: 3–4 points; very high risk: 5–7 points.
<7 umol/L	0	
>7 umol/L	3	
Pancreatic duct (PD)		
<5 mm	0	
>5 mm	2	
Shape of papilla		
Regular	0	
Irregular	2	

With the widespread use of various imaging modalities, the enlargement of the major duodenal papilla is increasingly being detected at CT. There are many reasons that could cause the enlargement of the papilla, such as papillitis, periampullary and ampullary cancer, pancreatitis, and choledochocele (1). And It is more difficult to identify the course when there was only enlarging duodenal papilla without obvious lesions in neighboring organization. The invasive operation and may be major post-procedural complications of ERCP prompt to find a non-invasive method to identify the pathological condition of the bulging papilla (6–8). Although CT can’t clarify the exact cause of enlargement, it can provide additional information such as dilatation of the common bile duct or PD, thereby could preoperatively predict of malignancy in the bulging duodenal papilla.

To focus on pathological abnormalities originated from the duodenal papilla, we excluded some conditions, such as stones in bile duct identified at CT or magnetic resonance imaging (MRI) and some lesions originated from pancreatic and common bile duct involving the major duodenal papilla.

We used lager than 5 mm at CT as the standard of the enlargement papilla. The sizes of the normal major duodenal papilla are various (1, 3). It described that the papilla was always less than 10 mm when identified by thin-section CT scans at some reports (1, 13, 14), but due to the volume effect, it may be inappropriate to use 10 mm as the standard for the enlargement of the major duodenal papilla. Therefore, we included patients with bulging duodenal papilla larger than 5 mm to avoid missing cases.

In our scoring system, DBil increase (>7 umol/L) is the only one clinical variable absorbed and the OR is 36.968 (95% CI 12.74–107.277) that weighted the highest score. For obstructive jaundice which can be frequently observed in patients with bulging duodenal papilla, some clinical characteristics has presented potential in the differential diagnosis of the benign and malignant cause like CA199 and total bilirubin (15–18). In this study, the normal range of TBil, DBil, IBil, and CA19-9 were 1.71–17.1 umol/L, 1.71–7 umol/L, 1.7–13.7 umol/L, and 0–37 Ku/L respectively. We found TBil increase (>17.1 umol/L), DBil increase (>7 umol/L), IBil increase (>13.7 umol/L), and CA199 increase (>37 Ku/L) was significantly different between benign and malignant bulging duodenal papilla, but only DBil increase (>7 umol/L) showed statistical significance in binary logistic regression, indicating the potentiality of this index.

**Figure 3:**
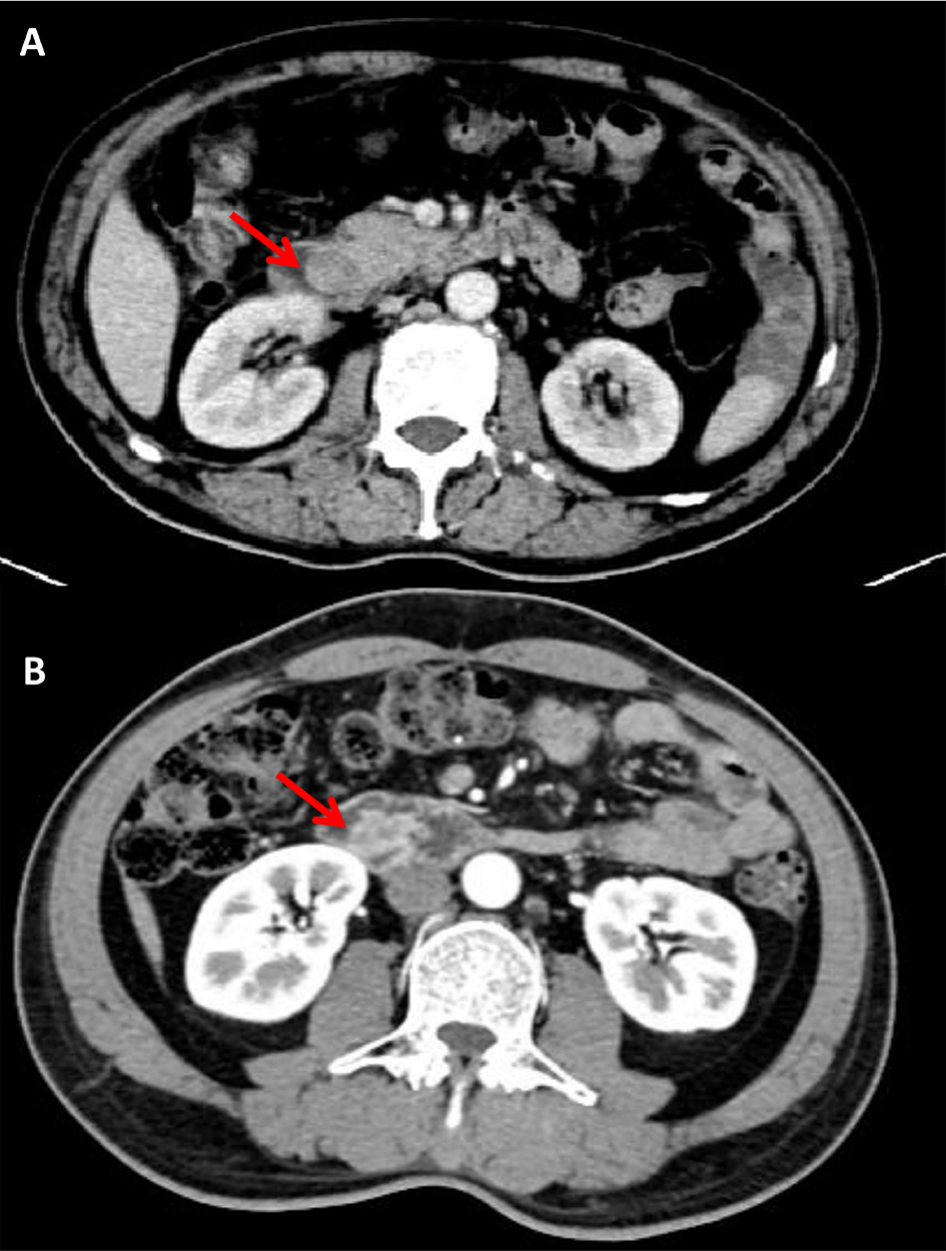
Benign enlargement of papilla in a 79 years old female. The shape of bulging papilla was regular (arrow) with a size of 18.6 × 19.7 mm **(A)**; Malignant enlargement of papilla in a 64 years old male, post-contrast image depicted irregular shape of bulging papilla with a size of 25.1 × 15.4 mm (arrow) **(B)**.

**Figure 4:**
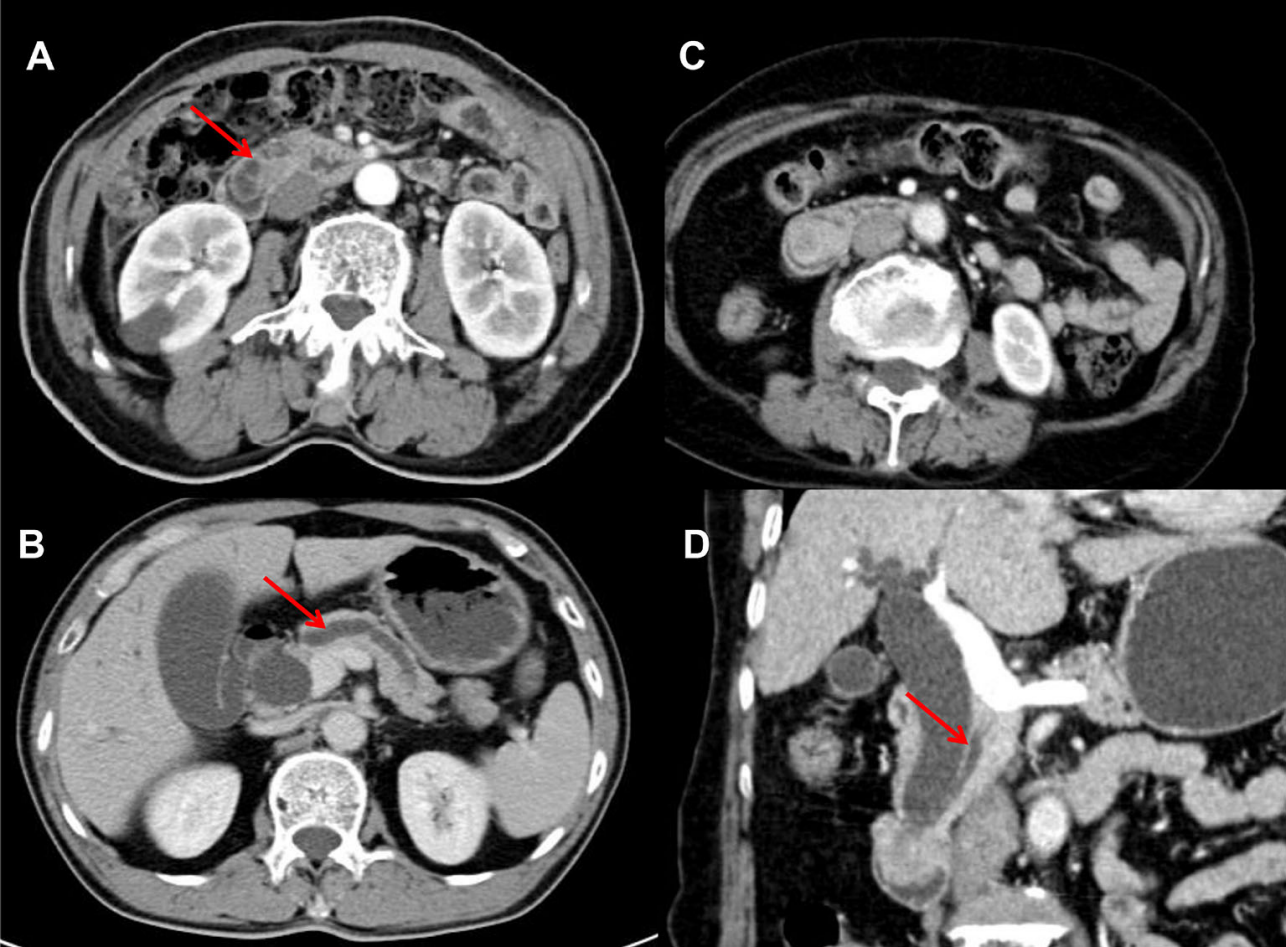
Malignant enlargement of papilla in 66 and 67 years old male respectively, post-contrast image depicted dilation of PD >5 mm (7.4 and 7.8 mm respectively) (arrow) **(A, B)**; Benign enlargement of papilla in a 75 years old female, the dilation of PD was 4 mm (<5 mm) (arrow) **(C, D)**.

Malignant lesions of the major duodenal papilla typically present as a hypoattenuating mass with enhancement on arterial and portal phase at CT (10) and its borders may be lobulated and infiltrating (19). The size of papilla/papillary mass was reported as the only independent variable to differentiated ampullary tumor from benign papillary stricture based on CT imaging (10). In our analysis, there was no statistical difference in the attenuation value of the two groups in the three phases or the size of papilla, but irregular shape of the bulging papilla was another variable of this scoring system (OR 7.435; 95% CI 1.73–31.953) (**Figure 3**). However, we mentioned that the appearance rate of irregular papilla was not high (10.2%) and it was more frequently seen when the papilla was relatively larger, suggesting that this characteristic may be more meaningful in this condition. Malignant papillary carcinomas often presented as small lesion when diagnosed because of the relatively early onset of symptoms, which may be difficult to be distinguished from other causes due to not obvious in images (20). In these condition, secondary findings, such as marked bile duct dilatation and mild to moderate dilatation of PD, can provide hints, which can be obviously presented in CT (21), and dilatation of both was seen in approximately 52% (13). The maximum diameters of this duct also showed potential in predicting malignancy (10). EHD >10 mm, EHD >20 mm, IHD >5 mm, PD >3 mm, and PD >5 mm all showed statistical difference between two groups, and only PD >5 mm was one of the independent variables in binary logistic regression (OR 8.403; 95% CI 2.509–28.14), reminding us pay attention to the degree of PD dilatation (**Figure 4**).

In previous studies, some other imaging features also had been reported. The enlargement of the major duodenal papillary caused by benign edematous thickening at the ampulla of Vater could present wall thickening and more intense enhancement than normal papillary (22, 23). We observed thickening of the adjacent duodenal wall and the distal of the common bile duct, and we found they both showed statistical difference between two groups. Although none of them were included in the logistic regression, both of these two conditions occurred in malignant enlargement of the duodenal papillary in this study, which still worthy of our attention.

There are some limitations in our study. Firstly, there may be inherent selection bias due to retrospective study design. Secondly, we wanted to further study the meaningful difference between patients with inflammation or diverticulum and with duodenal papillary adenoma, but there were no variables presented statistical significance in univariate analysis in this cohort, which indicating more patients and more variables may need to be brought in.

In conclusion, we established a scoring system for preoperative prediction of malignancy in the bulging duodenal papilla, which incorporating three critical variables, including DBil increase (>7 umol/L), PD dilation (>5 mm), and irregular shape of the bulging papilla. This scoring system has good discriminative ability for malignant enlargement of the papilla, and we believe that such prediction could have significant assistance in the clinical practice.

## Data Availability Statement

The original contributions presented in the study are included in the article/supplementary material. Further inquiries can be directed to the corresponding author.

## Ethics Statement

The studies involving human participants were reviewed and approved by the Second Affiliated Hospital of Zhejiang University School of Medicine. Written informed consent for participation was not required for this study in accordance with the national legislation and the institutional requirements.

## Author Contributions

The work reported in the above for publications has been done by all authors. X-JW contributed to data analysis and manuscript editing. J-LK collected the data of patients and J-XX supported. J-PZ, Y-FL, and DS helped in images analysis. Q-MZ helped in manuscript preparation. And R-SY contributed to the supervision of the whole process. All authors contributed to the article and approved the submitted version.

## Funding

This study was supported by funding from the Medical Science and Technology Project of the Health Department of Zhejiang Province of China (no. 2019334245).

## Conflict of Interest

The authors declare that the research was conducted in the absence of any commercial or financial relationships that could be construed as a potential conflict of interest.

## Abbreviations

PD, pancreatic duct; EHD, extrahepatic bile duct dilation; IHD, intrahepatic bile duct; CT, computed tomography; ERCP, endoscopic retrograde cholangiopancreatography; TBil, total bilirubin; DBil, direct bilirubin; IBil, indirect bilirubin; CA199, carbohydrate antigen199; ROC, receiver operating characteristic curve; AUC, area under curve.
